# Designing a Nanotube Using Naturally Occurring Protein Building Blocks

**DOI:** 10.1371/journal.pcbi.0020042

**Published:** 2006-04-28

**Authors:** Chung-Jung Tsai, Jie Zheng, Ruth Nussinov

**Affiliations:** 1 Basic Research Program, SAIC-Frederick, Inc., Center for Cancer Research, Nanobiology Program, National Cancer Institute-Frederick, Frederick, Maryland, United States of America; 2 Medical School, Tel Aviv University, Tel Aviv, Israel; Cornell University, United States of America

## Abstract

Here our goal is to carry out nanotube design using naturally occurring protein building blocks. Inspection of the protein structural database reveals the richness of the conformations of proteins, their parts, and their chemistry. Given target functional protein nanotube geometry, our strategy involves scanning a library of candidate building blocks, combinatorially assembling them into the shape and testing its stability. Since self-assembly takes place on time scales not affordable for computations, here we propose a strategy for the very first step in protein nanotube design: we map the candidate building blocks onto a planar sheet and wrap the sheet around a cylinder with the target dimensions. We provide examples of three nanotubes, two peptide and one protein, in atomistic model detail for which there are experimental data. The nanotube models can be used to verify a nanostructure observed by low-resolution experiments, and to study the mechanism of tube formation.

## Synopsis

Nanobiology is challenging to computational biology. The aim is to predict candidate nanostructures consisting of biopolymers. The idea the authors have followed for some years is to employ naturally occurring protein building blocks for protein and nanostructure design. Drawing on proteins as material is attractive, since proteins and their building blocks have a large repertoire of shapes and surface chemistry. The authors assume that the shape is given: either a target protein scaffold or a functional nanoparticle shape. Ideally, building blocks should self-assemble spontaneously. In practice, self-assembly involves time scales not affordable for computations. Instead, here is presented the first step in knowledge-based nanotube design: creating a nanotube with specified protein arrangement and tube geometry. Models are constructed by wrapping a planar sheet onto a cylinder surface. The sheet is shaped by a repeating 2-D lattice. This simplification reduces the complexity to the protein arrangement in the lattice and does not prevent construction of all possible nanotubes of repeated units. It allows optimization with all-atom force field. This is important since local energy minimization may screen whether a specified nanotube is a feasible nanostructure.

## Introduction

Designing a self-assembly nanodevice to perform a particular biological function is the ultimate goal for the upcoming era of nanobiology [1–4]. A nanodevice, to name a few, can be a drug delivery agent [5], a scaffold for tissue regeneration, or a biosensor [6,7] for detecting a toxic chemical or a particular biomarker. A designed nanodevice can contain one or more independent self-assembly biological nanostructures with a variety of different geometries, including 2-D bio-tapes [8], 2-D planar bio-sheets, bio-nanoparticles, bio-nanotubes, etc. Many self-assembled peptide and protein nanotubes have been recently observed experimentally. The nanotubes are observed as associated tubes in crystals [9,10], as embedded tube(s) in lipid membranes [11–13], as fused tubes of laminated amyloid fibrils [14], as branched network tubes in solution [15], or as isolated nanotubes in solution [16,17].

The idea that we have been following for a few years [18] is to employ naturally occurring protein building blocks for protein and for nanostructure design. We assumed that the shape is given: it is either the scaffold of a target protein or here, the shape of a predefined functional nanodevice. The strategy involves optimal mapping of candidate protein building block parts onto the nanostructure shape. The candidate building blocks were judiciously selected from a library of structures according to some criteria. If the conformation of the building block in the designed nanostructure is similar to that observed when it is embedded in the native protein, and its association with neighboring building blocks in the construct is favorable, the device has a chance to be stable. Here we address the very first step in the design process: how to perform the mapping of given folded building blocks onto the shape. Ideally, the building blocks would self-assemble. However, given the computational timeframes that are required, such a procedure is infeasible. Here, we choose the simplest shape, that of a nanotube. For building blocks, we selected cases for which there are experimental data that they form such a tube. The mapping led to atomic models of the isolated protein nanotubes.

**Figure 1:**
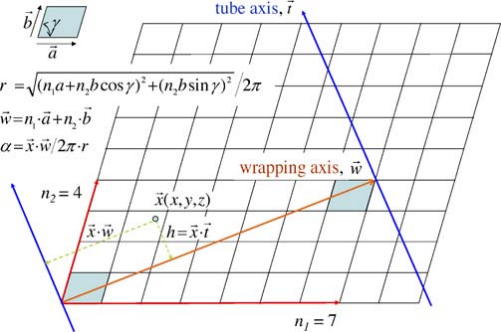
A Sketch to Illustrate the 2-D Lattice Wrapping System In the drawing, the 2-D lattice is highlighted in color with the angle *γ* between the two axes, and . The 2-D lattice wrapping process is drawn as an example of (7, 4) wrapping.

The tube construction procedure is like wrapping a planar sheet onto a tube surface [19]. It requires only five parameters for all possible arrangements of a building block on the tube surface if the arrangement has a 2-D repeating pattern. The planar sheet is shaped by a repeating 2-D lattice, which is described by three lattice constants *(a, b,* and *γ)*. How the planar sheet is wrapped onto the tube surface is defined by two wrapping integers *(n_1_, n_2_),* where *n_1_* states how many cells are used to wrap one full round along the lattice axis *a,* and *n_2_* indicates how many cells are shifted along the lattice axis *b* after one complete wrapping. A sketch to illustrate the five parameters is given in Figure 1. The detailed wrapping is described in the Methods section. The CHARMM 22 force field [20] was employed to optimize the tube structure under the 2-D lattice wrapping system with a local optimization method. The energy minimization is similar to that used in the optimization of a crystal structure under a periodic boundary condition. In the tube optimization, the periodic condition is replaced by the 2-D lattice wrapping system.

Constructing atomic models of nanotubes made from naturally occurring protein building blocks has many useful applications. Among these, it may help verify or eliminate a putative model proposed based on low resolution experimental evidence; it may help in understanding the mechanism of the tube formation [21,22]; and finally, it is definitely needed toward a total design of new nanostructure or nanodevice.

## Results

We applied the wrapping system to construct three protein nanotubes: a dipeptide (diphenylalanine) nanotube, a surfactant-like peptide (Ac-VVVVVVD) nanotube, and a protein (HIV-1 CA protein) nanotube. Below we provide the detailed construction processes for the three nanotubes.

### Diphenylalanine

Diphenylalanine has been reported to form long stiff nanotubes [16,17] and vesicles [16] with various sizes. However, under crystal growth conditions, diphenylalanine also forms crystals of associated nanotubes [10]. For each individual tube observed in the crystal, the tube is circled by merely six diphenylalanines with the peptide termini pointing toward the center of the tube and the two phenyl groups pointing outward. The crystal structure suggests that it should be feasible to create a nanotube of various sizes with the tube's thickness consisting of only two diphenylalanines. In such an arrangement, the two phenyl groups of diphenylalanine are on the same side of the backbone and point to the other two phenyl groups head-to-head. With these guidelines, we set out to construct the dipeptide nanotubes.

**Figure 2:**
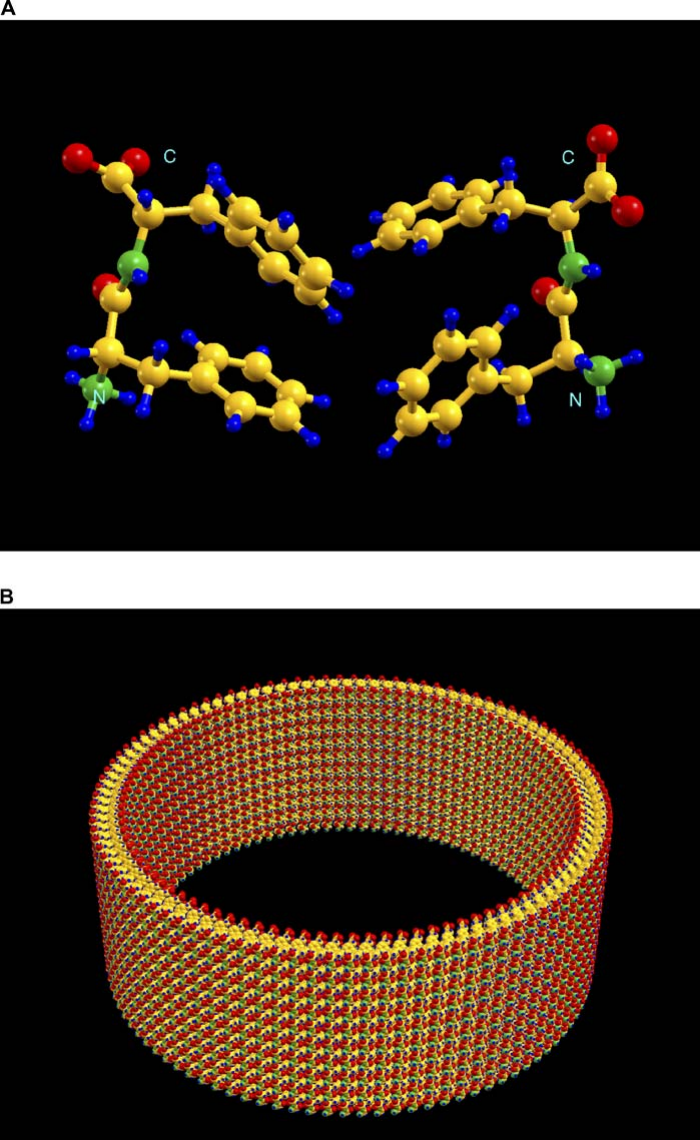
The Optimized Structure of the Diphenylalanine Nanotube (A) The structure of two diphenylalanine peptides in a lattice. The carbon atom is colored yellow, oxygen in red, nitrogen in green, and hydrogen in blue. (B) The nanotube constructed in 10 rounds with the optimized lattice constants (*a* = 6.8487, *b* = 8.7846, *γ* = 88.963). There are a total of 2,000 (100 × 10 × 2) diphenylalanine molecules in the constructed nanotube. The wall thickness of the tube is around 16 Å. The inner wall diameter of the tube is 202 Å and the outer wall diameter is 234 Å.

The initial structure of the diphenylalanine was created from the backbone-dependent rotamer library in an α-helix conformation. The monomer was next manipulated through the rotatable bonds to have the two phenyl groups pointing from the same side of the backbone. After the two monomers were created with the phenyl groups pointing toward each other via the graphic tool, a local optimization was performed with the following five wrapping parameters *(n_1_, n_2_, a, b,* and *γ):* 100, 0, 7.0, 9.0, and 90.0. This gave the initial tube a radius of 111.408 Å and tube wall thickness of about 16 Å. The lattice constants of the optimized tube were *a* = 6.8487, *b* = 8.7846, and *γ* = 88.963, which are pretty close to the initial values. The optimized CHARMM energy of the two diphenylalanines in the lattice was −227.8887 kcal/mol. The optimization process took only 25 computer processing unit s with a Linux box running on Intel Xeon 3.0 GHz. The structure of the two diphenylalanine molecules in a lattice after optimization is given in Figure 2A. The nanotube, which was constructed in ten complete rounds with a total of 2,000 (100 × 10 × 2) diphenylalanine molecules, is given in Figure 2B.

The experimental electron microscopy (EM) images [16] indicated that the typical diphenylalanine nanotubes had an outer diameter of 4,000 to 20,000 Å, an inner diameter of 3,000 to 18,000 Å, and a wall thickness of 500 to 2,000 Å. The EM data suggested that the wall thickness needed to increase 35 to 143 times and the radius of the tube needed to enlarge at least 13 times for the tube constructed above. To adjust a tube's radius is straightforward with the current wrapping system. It is simply done by increasing or decreasing the value of *n_1_* to reflect the size requirement. By increasing *n_1_* from 100 to 1350, we should have had a corresponding tube radius as seen in the EM image. For escalating the wall thickness, we could duplicate the diphenylalanine dimer along the *z*-axis (or perpendicular to the tube axis) into a linear tetramer to double the wall thickness. Repeating the thickness-doubling procedure five times, the wall thickness should be very close to the experimental data. The details of constructing such a tube with size compatible to the experimental size will be given elsewhere.

### Ac-VVVVVVD

The Ac-VVVVVVD (V_6_D), a surfactant-like peptide with an acetylated N terminus, as well as many other similar surfactant-like peptides, have been observed to form a network of open-ended nanotubes and vesicles [15,23]. The lipid-like structural feature, one end with charged group and the other all hydrophobic moieties, suggests that the wall of the tube or vesicle is lined up with two monomers head-to-head just like the lipid arrangement in the membrane. The head-to-head arrangement is also supported by the EM image reflecting a tube wall thickness of around 40 Å, corresponding to the length of two head-to-head peptides in extended conformation. Therefore, the surfactant-like peptide nanotube is constructed in a way that is very similar to the one suggested by Vauthey et al. [15]

**Figure 3:**
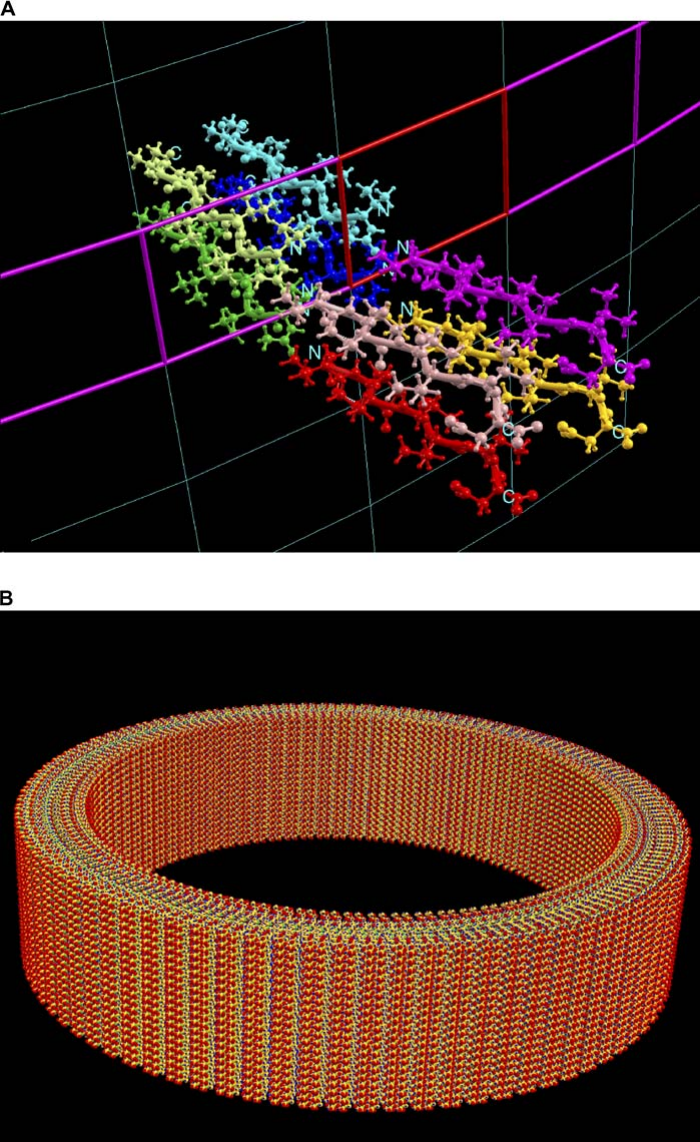
The Structure of the Ac-VVVVVVD Nanotube (A) The initial conformation of the eight surfactant-like peptides. Each peptide is highlighted in different colors with N terminus pointing head-to-head. (B) The optimized Ac-VVVVVVD nanotube built in ten rounds with the charge–charge interaction being turned off. There are a total of 6,800 (85 × 10 × 8) peptides in the built nanotube. The wall thickness of the tube is around 49 Å. The inner wall diameter of the tube is 407 Å and the outer wall diameter is 505 Å.

The initial structure of V_6_D was created from the backbone-dependent rotamer library in the β-strand conformation. Instead of just using two monomers to construct the tube, eight monomers with acetylated N termini were manipulated via the graphics tool to arrange them in a head-to-head orientation. The distance between the peptides was next adjusted graphically as close to each other as possible with limited collision between sidechain atoms. With the eight peptides sitting in a 2-D lattice of *a* =17, *b* = 12, and *γ* = 90.0, the two wrapping constants *(n_1_, n_2_)* were set to (85, 0) in order to have an initial tube radius of about 230 Å to match the experimental outer diameter (500 Å) of the V_6_D tube. The initial conformation of the eight peptides, ready for local optimization, is depicted in Figure 3A. After optimization, the cell dimension expanded (*a* =19.36, *b* = 16.56, and *γ* = 77.98) and the radius of the tube increased from 230 to 261.9 Å. This indicated that the created protein arrangement in the initial structure might not be stable. The optimized result revealed that peptides in the tube seem unable to satisfy their electrostatic interactions under the CHARMM 22 force field in vacuum, especially the interactions at both termini. The unsatisfied electronic interactions were verified by the fact that an optimized structure did maintain the initial cell dimension (*a* =16.88, *b* = 11.85, and *γ* = 90.0) when the charge–charge interaction is turned off in CHARMM. The optimization took 105 computer processing unit min. The optimized CHARMM energy was −183.0030 kcal/mol without electrostatic terms. Figure 3B gives the constructed nanotube in 10 complete rounds with a total of 6,800 (85 × 10 × 8) peptides. Since we did not use a force field in explicit water, the contribution from electrostatic interactions may not have been accounted for accurately. We were therefore unable to either confirm or reject the model suggested by Vauthey et al. [15]

### HIV-1 CA protein

In vivo, the CA protein from the HIV-1 typically forms as a conical capsid. In vitro, the CA protein spontaneously assembles either into a tube or cone. A recurring hexameric ring has been observed on all tubes. An atomic model with the CA protein arranged with approximate local P6 lattice symmetry has been established by docking crystal structures of the amino-terminal domain (NTD) and the carboxyl-terminal domain (CTD) into the reconstructed density from the EM image [24]. The docking result reveals that the hexameric ring is the outcome of the association between six NTDs and suggests that dimerization of two CTDs connects each ring to six neighbors. The model clearly shows that the ring is connected by a 12-helix bundle with two helices (Helix-1 and Helix-2) from each NTD. However, the association between two CTDs is not precisely defined from the docking result.

**Figure 4:**
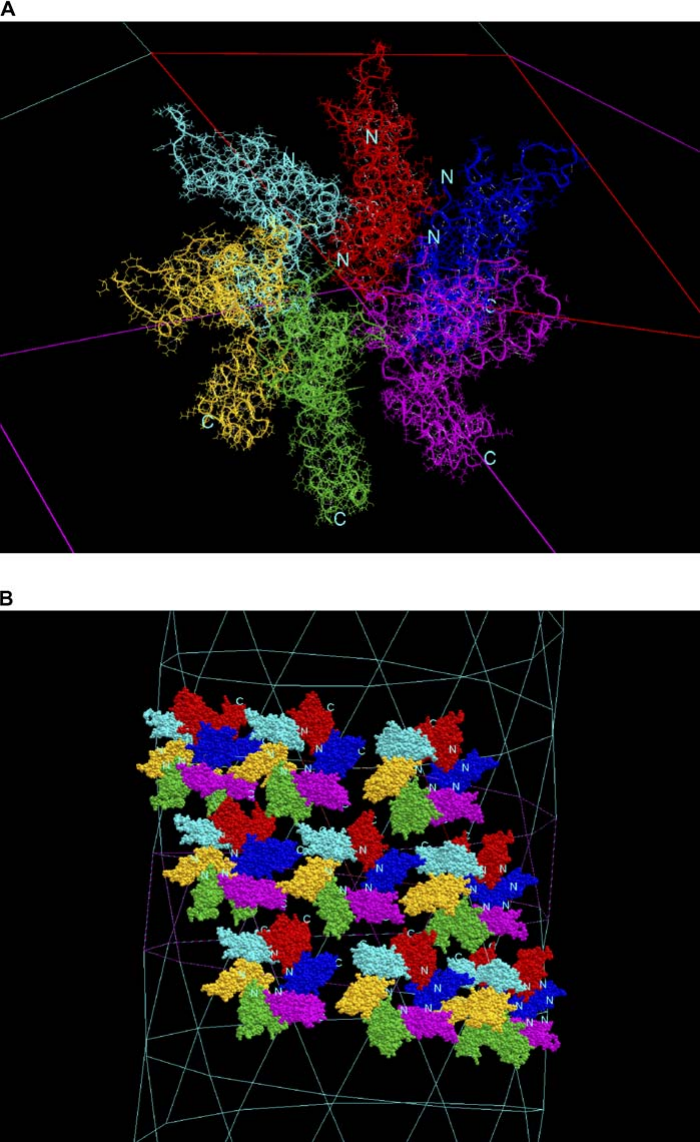
The Structure of the HIV-1 CA Protein Tube (A) The initial conformation of the hexameric ring with NTD sitting on the outer wall of tube. Each CA monomer is highlighted in different color with backbone trace in ribbon. The ring is connected by a 12-helix bundle with two helices contributed from each NTD. (B) Nine copies of the hexameric ring sitting on the optimized CA protein tube.

In the CA protein nanotube construction, the initial structure of the CA protein was taken from the crystal structure [25] available in the Protein Data Bank [26] (PDB code 1e6j, chain P). The CA crystal structure contains both the NTD and the CTD domains. The two domains are connected by a flexible linker of six residues. Following the EM images, we constructed the tube by assigning the 2-D lattice constants (108.0, 108.0, 120.0) with the P6 lattice symmetry and two wrapping constants of (12, 1). The crystal coordinates were manipulated, translating and rotating the molecules to form a hexameric ring. At the center of the ring was a 12-helix bundle composed of Helix-1 and Helix-2 from six NTDs generated by the P6 lattice symmetry. At this point, without any further modification to the structure, the CTD did not have any contact with other CTDs. The initial structure is given in Figure 4A. After energy minimization, the optimized tube gave a CHARMM energy of −3306.66 kcal/mol for one CA protein with a little shrunken cell (106.4596, 106.4596, 120.0). The optimization took 23 computer processing unit h. The CA protein tube is shown in Figure 4B with nine copies of the hexameric ring. The optimized tube did not give the association as seen in a crystal CTD dimer [27] or implied by some mutation data on attenuating or enhancing tube formation [28]. However, we were now in a position to construct a CA protein tube in accordance with all experimental data.

## Discussion

**Figure 5:**
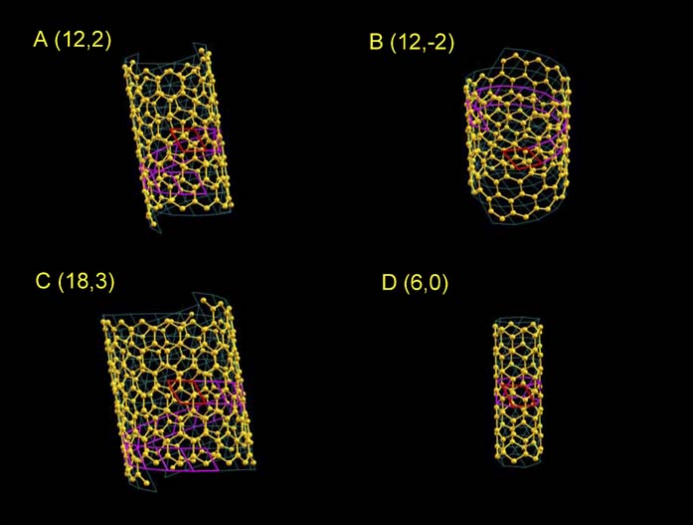
Construction of Carbon Nanotubes with Different Parameters Four carbon nanotubes with different wrapping constants *(n_1_, n_2_)* are constructed with the 2-D lattice (*a* = 2.459512, *b* = 2.459512, *γ* = 120), P6 lattice symmetry, and one-carbon fractional coordinates (1/3, 2/3, 0). In each nanotube, there are *n*_1_*7 cells shown in cyan with the origin of the lattice highlighted in red and one complete wrapping round in magenta.

There is an inherent geometrical distortion when wrapping a planar 2-D lattice onto the surface of a cylinder. The distortion is an inevitable outcome of the geometrical transformation. After wrapping, the planar 2-D lattice is no longer planar; rather, it is a twisted 2-D lattice. The carbon nanotubes depicted in Figure 5 are good examples to illustrate the distortion. In graphite, the carbon–carbon bond distance of the planar structure is always a constant (1.4200 Å used here). There were three sets of distinct bond distances that were formed after the planar structure is wrapped to form a nanotube. For the (12, 2) nanotube, the three carbon–carbon bond distances were 1.4177, 1.4152, and 1.4200 Å. The (12, −2) nanotube gave 1.4167, 1.4182, and 1.4200 Å. The (18, 3) nanotube had 1.4190, 1.4179, and 1.4200 Å. The (6, 0) nanotube exhibited 1.4079, 1.4079, and 1.4200 Å. These distorted distances clearly show that the distortion is inversely proportional to the radius of a nanotube, with the (18, 3) tube having the lowest distortion and the (6, 0) tube the highest. If the amount of distortion was measured for the atoms exactly on the tube surface, it is defined here as “distortion at the tube surface.”

In addition to the distortion at the tube surface, there was another wrapping distortion if a 3-D structure instead of a planar sheet was wrapped onto a tube. For the wrapping system described above, the wrapping process would enlarge the portion of the 3-D structure that is above the planar sheet (or outside the tube after wrapping). On the other hand, the portion of the 3-D structure that is below the planar sheet (or inside the tube after wrapping) would shrink. We call this kind of distortion “perpendicular distortion.” The distortion resembled a scale up or down of the distortion on the tube surface, which is more severe than expected. The magnitude of the “distortion at the tube surface” was proportional to the curvature of the constructed tube. The magnitude of the “perpendicular distortion” is determined by the wall thickness, or more precisely, it is proportional to the distance to the tube surface and the curvature of the tube. The extent of the 3-D wrapping distortion can be roughly judged by the ratio of the wall thickness over the tube radius, with a larger number implying larger distortion. Among the three constructed tubes, the Ac-VVVVVVD nanotube exhibited much higher visible molecular distortion than the other tubes. To prevent both distortions in a molecule, we might superimpose the molecule before wrapping onto the distorted molecule after wrapping and then replace it. This is referred to as molecular distortion correction.

In principle, nanotube construction with molecular distortion correction should create an atomic model closer to that of an actual, realistic nanotube. However, with or without molecular distortion correction, the tube constructed here always displayed a higher density toward the tube center and a lower density away from the tube center. This fact explains why the diameter of an observed protein nanotube always has a lower and upper limit. If the diameter of the tube is too small, the portion inside the tube will be too crowded. On the other hand, the packing of the outside part of the tube will be too loose to hold the structure.

For the tube construction, the two wrapping integers were always predefined and kept unchanged. They implicitly determined the size of the tube and the arrangement of the tube skeleton. In principle, an available EM image could provide hints for the assignment of appropriate wrapping constants. In de novo nanotube design, however, we had to face the problem of how to determine the two appropriate wrapping constants. Here we used an ad hoc coarse-graining procedure to assign the two wrapping constants. Starting with *n_2_* = 0, we determined the best value for *n_1_.* Then, we found the best value for *n_2_* with the already determined *n_1_.*

Since the potential energy landscape of a constructed protein nanotube in terms of the CHARMM 22 force field is rugged, it is not surprising that a slightly different initial conformation always gives a distinct optimized structure. Even though a locally optimized structure is not unique in many respects, the nanotube construction is considered completed only after an energy minimization is performed. An optimized nanotube structure usually can reveal its overall stability through structural evaluation in terms of hydrophobicity, compactness, and satisfied electrostatic interactions. Hence, a local energy minimization acts as the first screen in estimating whether a constructed protein nanotube is a feasible nanostructure. For the Ac-VVVVVVD, the results of the optimization during the construction of the nanotube raised a question as to its feasibility.

In this study, the tube optimization was executed only by a local optimization method with the CHARMM 22 force field in vacuum. In the future, a global optimization method, the parallel tempering molecular dynamics (MD) method, will be utilized to prevent a structure from being trapped on a rugged potential surface during the optimization. The constructed protein nanotube should be located at the bottom, rather than on the side of the potential energy well. If at the bottom, the results of the constructed structure will be more representative. This will also lessen the impact of the guess of an initial conformation, which is currently required to be very close to that of the expected optimized structure. Further, the CHARMM 22 force field in the explicit water model was much more realistic than the model in vacuum. With the 2-D periodic condition constructed with the 2-D wrapping system, it is feasible to simulate a constructed nanotube using MD to test its stability in explicit water. Explicit water MD simulations can be performed on a constructed tube with two layers of water molecules, one inside and the other outside the tube.

Establishing an atomic model for a protein nanotube is useful in many ways. First, it can be used to confirm or reject a putative model based on some (low-resolution) experimental data. For example, if a constructed nanotube is unstable, the chance that the tube will form in that particular way is low. Second, an atomic model should provide valuable information regarding the mechanism of the tube formation. For instance, the comparison between a constructed nanotube and a planar model may help in explaining why a tube or a vesicle (void sphere) has a tendency to curve. Third, the existence of an atomic model whose features are verified by experiment should boost the utility of the experimental results for various biological applications. Fourth, this is the first step toward a de novo protein nanotube design. From the theoretical point of view, the complete process of designing a new protein nanotube can be divided into three stages. The first stage is to construct a specified nanotube from a given peptide or protein. The second is to verify that the constructed tube is a stable structure. The third stage involves computational validation that the designed tube is the major product during the process of self-assembly.

Given the current limitations of the computational resources and the accuracy of the molecular mechanics force field, it is infeasible to carry out ab initio calculations in an attempt to self-assemble a nanostructure automatically. However, it is achievable to construct, rather than predict, an atomic model by incorporating any available experimental data such as images from EM. Here, a constructed atomic nanostructure serves two main purposes. First, we may ask if a constructed nanostructure truly supports all available experimental data. If yes, the established atomic model will be valuable for further design toward a nanodevice. If no, the discrepancy between the constructed model and experimental data will be useful for the next round of construction. Second, in order to design a nanostructure with a specified geometry, the capability of constructing such a designed nanostructure is the very first step.

In this paper, we only focus on the first stage of how to construct a tube from its protein building blocks for evaluation in later stages. Even so, it is important to address the question if the self-assembly can really take place to form the constructed candidate. Otherwise, the whole design strategy seems less valuable. We believe that a self-assembly can be predicted to happen if and only if the associations between building blocks are the most popular states. This proposition further highlights another advantage of our approach since under these circumstances only a limited number of associations are introduced into the entire tube under the 2-D lattice wrapping system. We only need to check if those associations that form in the constructed model are among the most favorable interactions between the building blocks. For example, even in the simplest case here, that of the diphenylalanine nanotube, the tube construction is not trivial since all favorable interactions were considered and introduced in the model. The constructed tube can be used as a foundation for building an atomic model with the tube geometry as observed by experiments. Since computationally it is currently infeasible to obtain a definitive model of the self-assembly with the presently available all-atom force field, any suggested theoretical model still needs to be verified by experiment.

The current work is within the framework of our ongoing effort to design protein and nanostructures using protein building blocks [18,29]. The Protein Data Bank is populated by an immense number of naturally occurring proteins and their parts, with a rich repertoire of shapes and chemistry [30]. Our goal is to use the pool of candidates, selecting those that conform to certain criteria, and string them together to build modules. If the conformational preference of the building block is retained within the module, and its association is favorable, the nanostructure may be stable. Since it is infeasible to let the building blocks self-assemble, it is essential to have a procedure to map them onto the desired shape. Here we chose the simplest shape, that of the nanotube. Our strategy involved mapping the building blocks onto a planar sheet, which is shaped by a repeating 2-D lattice and wrap the lattice onto the tube surface. We applied the procedure to three different types of protein nanotubes, a Phe-Phe aromatic dipeptide, a hydrophobic surfactant, and the CA protein of the HIV-1 virus capsid, where there are some reference experimental data.

Modeling nanostructures has many applications. Among these, from the biological standpoint, is the availability of detailed atomic scale models for experimentally obtained nanostructures whether designed or obtained unexpectedly. These allow modifications and additional cycles of design. In addition, a verified atomic model can be used toward the goals of a self-assembled designed nanodevice and for studies of the mechanism of tube formation.

## Materials and Methods

### 2-D lattice wrapping system.

The 2-D lattice wrapping system described below can construct all possible as well as any specified symmetrical arrangements that a nanotube can form. Only five parameters *(n_1_, n_2_, a, b,* and *γ)* are needed for the 2-D lattice wrapping system to construct the framework of a tube. The first two wrapping integers *(n_1_, n_2_)* are used to define how a 2-D lattice is wrapped around the tube. The number *n_1_* states how many cells are used to wrap one full round along the lattice axis 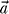 *,* and the number *n_2_* indicates how many cells are shifted along the lattice axis 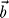 after one complete wrapping. The angle between the two axes of the lattice is *γ*. If we let the lattice lie on the Cartesian *x–y* plane and let the axis 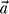 lie along the Cartesian *x*-axis, then the vector of lattice axis 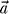 in Cartesian coordinates is *(a, 0, 0),* and the vector of lattice axis 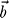 is *(bcosγ, bsinγ, 0).* The wrapping definition above delineates the vector of the wrapping axis 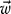 as *n_1_(a, 0, 0)* + *n_2_(bcosγ, bsinγ, 0)*. The axis of tube 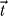 can be obtained by rotating the wrapping axis by 90° via the Cartesian *z*-axis. The radius of the tube, *r,* is then calculated as [(*n_1_a*+*n_2_bcosγ*)^2^ + (*n_2_bsinγ*)^2^]^1/2^ / 2π. A sketch of the 2-D lattice wrapping system is given in Figure 1.

The actual wrapping calculation was done as follows. First, we projected given Cartesian coordinates 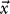 *(x, y, z)* onto both the wrapping axis 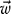 and the axis of tube 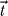 to find out the rotation angle α and the shifting distance *h* along the axis of the tube after rotation. The rotation angle and the shifting distance were calculated as α = 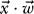 /*2πr*; *h =* 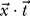 *.* Second, a rotation matrix was calculated with the rotation angle α and the rotation axis 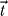 . Third, the point at *(0, 0, z +r)* was rotated by the rotation matrix. Finally, the wrapping coordinates were obtained by shifting the previously rotated point by a distance of *h* along the tube axis 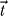 .

Since the symmetry operations in a 2-D lattice are applied to the fractional crystallographic coordinate 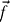 *(f_x_, f_y_),* not the Cartesian coordinate, interchanges between the two coordinate systems were needed. This was done by 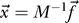 and 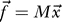 , where the orthogonalization matrix *M^−1^* and deorthogonalization matrix *M* were


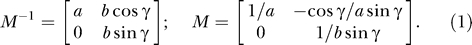


### Tube construction.

Given five wrapping parameters *(n_1_, n_2_, a, b,* and *γ),* a space group for the 2-D lattice, and an object in the asymmetric unit, the entire tube could be constructed as follows. First, if the objects were given in Cartesian coordinates we converted them into fractional coordinates with the orthogonal transformation. Second, all symmetric units within the lattice were generated according to the given 2-D space group. Third, we registered all of the cells according to the specified portion of the tube that was to be built. Each registered cell had the cell coordinates (*m*_1_, *m*_2_), with their cell positions relative to the origin cell coordinates (0, 0). Finally, to finish the tube construction we iteratively generated the coordinates for each registered cell. For each registered cell, we first shifted the fractional coordinates by adding the cell coordinates (*m*_1_, *m*_2_) to the fractional coordinates. The shifted fractional coordinates of the cell were converted back to Cartesian coordinates by the deorthogonal transformation. At the end, the tube Cartesian coordinates within the cell were calculated according to the wrapping transformation described above.

For simplicity, we used the example of a carbon nanotube to illustrate the tube construction procedure. The data for the carbon nanotube construction are: the 2-D lattice constants (2.459512, 2.459512, 120), the space group with P6 lattice symmetry, and one-carbon fractional coordinates (1/3, 2/3, 0). The length of the lattice was given to meet the distance of carbon–carbon covalent bond of 1.42 Å. Four carbon nanotubes constructed with different wrappings (*n*_1_, *n*_2_) are shown in Figure 5A–D. Each nanotube was specified to be constructed in seven wrapping rounds. Due to the P6 symmetry which required *a = b* and *γ = 120 °,* only three independent parameters were required to construct a carbon nanotube. In the figure, the framework of a tube is shown in *n*_1_*7 wrapping cells in cyan color with the origin of the lattice highlighted in red and one complete wrapping round in magenta. In each cell, there are two carbon atoms with the additional carbon generated by the P6 symmetry operation.

In Figures 5A and 5B, 12 cells are shown to complete one wrapping round with a shift in two cells, corresponding to the absolute value of the second wrapping integer (12, ±2). The (12, 2) nanotube in Figure 5A with a positive wrapping integer shows that the wrapping is a left-handed helix. On the other hand, the (12, −2) nanotube in Figure 5B with a negative integer, gives a right-handed wrapping helix. Figures 5C and 5D illustrate that the radius of the tube is implicitly proportional to the first wrapping integer and the second wrapping integer controls how a 2-D lattice wraps around the tube. The size of the (18, 3) nanotube was roughly triple the size of the (6, 0) nanotube, whose size in turn was about half of the size of both the (12, ±2) nanotubes. The (6, 0) nanotube with a zero second wrapping integer shows the stacking of circular wrapping.

### Local optimization.

The 2-D wrapping system described above enabled us to optimize the structure of the entire constructed tube with the independent variables, including only the fractional atomic coordinates in the asymmetric unit plus the three 2-D lattice constants. The two wrapping constants were kept unchanged during the energy minimization process. The optimization was similar to a full crystal energy minimization under a periodic boundary condition. In crystal optimization, it is straightforward to set up the periodic boundary conditions. For tube optimization, the periodic boundary conditions were set up according to the wrapping system described above. All optimizations were performed under the CHARMM 22 force field. For local optimization, either the NVT MD (in NVT MD, the number of particles *N,* the volume *V,* and the temperature *T* are kept constant) with the temperature set to zero or the OCVM method [31] (optimally conditioned variable metric nonlinear optimization without line searches) were employed. In the NVT MD method, the 2-D lattice has to be kept fixed. On the other hand, the OCVM method can optimize concurrently both the coordinates and the lattice constants. The gradients with respect to the CHARMM force field is calculated analytically and the gradients with respect to the lattice constants are calculated numerically. The CHARMM energy and force were truncated using the force switch and the van der Waals shift method with cutoff distance of 8 and 10 Å. The two TINKER 4.2 source codes [32], ocvm.f and xtalmin.f, helped us substantially in the coding of the local optimization integrated into the program “mcmd.” This program has been used to study the energy landscape of oligomerization of an amyloidogenic peptide with parallel tempering MD [33]. The optimization can be done in vacuum or in the explicit water model. In this paper, we only report the results of optimization with CHARMM 22 force field in vacuum.

### Initial conformation.

For a folded protein, the initial atomic conformation was taken from the Protein Data Bank [26]. For short peptides, we applied an in-house database of backbone-dependent sidechain rotamer library to create an initial conformation, either an α-helix or a β-strand. The initial position relative to the specified 2-D lattice was manipulated by an in-house graphics program to create a reasonable starting point. In addition, we also adjusted the protein conformation, if needed, through rotatable bonds to a suitable starting structure via the graphics program. The initial value of the two wrapping integers and the three lattice constants were either taken from the literature as determined by experiments if available, or are specified by a reasonable assignment.

## Abbreviations

CTD: carboxyl-terminal domain
EM: electron microscopy
MD: molecular dynamics
NTD: amino-terminal domain
